# Impact of Exendin-4 on the Differentiation and Function of Transplanted Porcine Neonatal Pancreatic Cell Clusters in Diabetic Nude Mice

**DOI:** 10.1155/jdr/5847576

**Published:** 2025-10-17

**Authors:** Jyuhn-Huarng Juang, Chen-Yi Chen, Chen-Wei Kao, Chen-Ling Chen, Wan-Chun Li

**Affiliations:** ^1^Division of Endocrinology and Metabolism, Department of Internal Medicine and Center for Tissue Engineering, Chang Gung Memorial Hospital, Taoyuan, Taiwan; ^2^Department of Medicine, College of Medicine, Chang Gung University, Taoyuan, Taiwan; ^3^Institute of Oral Biology, College of Dentistry, National Yang Ming Chiao Tung University, Taipei, Taiwan; ^4^Department of Dentistry, College of Dentistry, National Yang Ming Chiao Tung University, Taipei, Taiwan; ^5^Oral Medicine Innovation Center (OMIC), National Yang Ming Chiao Tung University, Taipei, Taiwan; ^6^Department of Stomatology, Taipei Veterans General Hospital, Taipei, Taiwan

**Keywords:** differentiation, exendin-4, glycemic control, porcine neonatal pancreatic cell clusters, transplantation

## Abstract

Porcine neonatal pancreatic cell clusters (NPCCs) are a promising and abundant source for islet transplantation owing to their ability to secrete insulin and proliferative potential. However, a significant limitation is the delayed normalization of blood glucose following transplantation, largely attributed to incomplete *β*-cell differentiation. Exendin-4, a glucagon-like peptide-1 receptor agonist, has been shown to enhance *β*-cell differentiation, proliferation, and function in various models. This study was aimed at investigating whether posttransplant treatment with exendin-4 could accelerate NPCC differentiation and improve long-term glycemic outcomes in diabetic nude mice. NPCCs were isolated from 1- to 3-day-old piglets and transplanted under the kidney capsule of streptozotocin-induced diabetic nude mice. Recipients received subcutaneous injection of either exendin-4 (3 *μ*g/kg) or saline (as control) twice daily. Graft differentiation was assessed at Days 6, 9, and 16 via immunostaining for insulin, glucagon, PDX1, and SOX9. Blood glucose levels, body weight, and graft histology were monitored over time. Exendin-4 treatment significantly increased the proportion of mature insulin^+^ cells at Day 9 (*p* < 0.05) and both PDX1^+^/insulin^−^ and SOX9^+^ progenitor cells at Day 6 (*p* < 0.01). By Week 10, hyperglycemia persisted in both groups; however, exendin-4-treated recipients exhibited a significant reduction in blood glucose from Week 6 onward. At this time point, grafts from hyperglycemic mice displayed both insulin^−^/PDX1^+^ (progenitor cells) and insulin^+^/PDX1^+^ (mature) *β*-cells, indicating ongoing differentiation. Among mice maintained beyond 10 weeks, euglycemia was achieved between Days 119 and 308, with grafts exhibiting mature insulin^+^/PDX1^+^ cells—evidence of morphological and functional maturation. Our results indicate that exendin-4 transiently enhances NPCC differentiation and expands the progenitor population in early posttransplantation phases. Furthermore, it contributes to improved graft performance and glycemic control, likely through its well-established metabolic effects.

## 1. Introduction

Most successful cases of human islet transplantation need multiple transplants to achieve insulin independence in patients with Type 1 diabetes [1–3]. Unfortunately, the number of available pancreases is far behind the ever-growing number of patients on the waiting list for islet transplants. To overcome this supply problem, xenogenic islets from animal sources are considered. Porcine neonatal pancreatic cells clusters (NPCCs) isolated from 1- to 3-day-old pigs, which are easily isolated and capable of secreting insulin and have growth potential, have been utilized for the treatment of diabetic rodents [4–7], pancreatectomized pigs [8], and nonhuman primates [9]. However, it took several weeks to cure diabetes after transplantation of NPCCs. Insufficient mature beta-cells are one of the reasons for delayed normalization of glycemia after transplantation [5]. Investigators have tried to enhance NPCC maturation by culturing them in media with growth factors, but this approach did not result in faster achievement of normoglycemia after transplantation [6, 7].

Glucagon-like peptide 1 (GLP-1) is an intestine-derived insulinotropic hormone that stimulates glucose-dependent insulin production and secretion from pancreatic beta-cells, suppresses glucagon secretion, delays gastric emptying, reduces appetite and food intake, and promotes glucose uptake in peripheral tissues [10]. All of these actions are potentially beneficial for the treatment of Type 2 diabetes mellitus. Moreover, GLP-1 was shown to stimulate the growth and differentiation (neogenesis) of *β*-cells [11] as well as inhibition of *β*-cell apoptosis [12, 13]. Exendin-4, a GLP-1 receptor agonist resistant to dipeptidyl peptidase-IV-mediated inactivation, exhibits effects similar to GLP-1 [10, 14–16]. In diabetic recipients transplanted with a marginal number [17, 18] instead of sufficient number [19] of adult mouse islets, posttransplant exendin-4 treatment improved metabolic control and expanded graft *β*-cell mass. Notably, exendin-4 treatment promoted the graft function and increased *β*-cell number in diabetic recipients transplanted with mouse and human fetal islet-like cell clusters [20, 21].

Our previous study demonstrated that NPCCs differentiate into mature *β*-cells at 9 and 16 days after transplantation in diabetic mice [22]. Therefore, in the present study, we applied this model to examine whether posttransplant exendin-4 treatment is beneficial for the differentiation and long-term function of transplanted NPCCs.

## 2. Materials and Methods

### 2.1. Animals

The animal experiment protocol was approved by the Institutional Animal Care and Use Committee (IACUC, No. 2019091802) of Chang Gung Memorial Hospital, Taiwan. Donor pancreases were harvested from neonatal pigs aged 1 to 3 days, regardless of sex. Male athymic nude BALB/c mice (8–12 weeks old) were used as recipients for NPCC transplantation. Diabetes was induced by a single intraperitoneal injection of streptozotocin (STZ; Sigma Immunochemicals, St. Louis, MO, United States) at a dose of 190 mg/kg body weight, administered 14 days prior to transplantation [7, 22]. All mice were housed under specific pathogen-free (SPF) conditions in an accredited animal facility. Mice were maintained in individually ventilated cages with a 12-h light/dark cycle, ambient temperature of 22°C ± 2°C, and relative humidity of 40%–60%. Animals had free access to autoclaved standard rodent chow and sterilized drinking water. The animal numbers, for both pigs and nude mice, used in the study are described in Table 1.

### 2.2. Isolation and Culture of NPCCs

Donor pancreases were cut into small fragments (approximately 1–2 mm^3^) and enzymatically digested using collagenase type V (Sigma Immunochemicals) in a 37°C water bath. The digested tissue was then filtered, washed, and cultured in RPMI medium under standard conditions (37°C, 5% CO₂, 95% air, and humidified atmosphere) [7, 22]. The characteristics of isolated NPCCs have been described previously [23].

### 2.3. NPCC Transplantation

After 3 days of in vitro culture, 2000 NPCCs were transplanted into each diabetic nude mouse. The NPCCs were centrifugated into PE-50 tubing connected to a 200-*μ*L pipette tip. A small incision (capsulotomy) was made in the lower pole of the kidney, and the tubing tip was inserted under the renal capsule at the upper pole for cell injection [7, 22]. Blood glucose levels and body weights were monitored regularly following transplantation. Normoglycemia was defined as a nonfasting blood glucose level below 200 mg/dL.

### 2.4. Exendin-4 Treatment

Following NPCC transplantation, recipient mice received subcutaneous injections of exendin-4 (3 *μ*g/kg; Sigma Immunochemicals) or normal saline twice daily [18].

### 2.5. Immunofluorescence Staining of the Islet Graft and Quantitative Histological Analysis

The removed grafts were fixed in formalin solution and processed for paraffin embedding and sectioning. Sections of grafts were stained for the endocrine cells with immunofluorescence by guinea pig anti-insulin antibody (Abcam, Cambridge, United Kingdom), rabbit anti-glucagon antibody (Dako Inc., Carpinteria, CA, United States), rabbit anti-PDX1 antibody (Abcam, Cambridge, United Kingdom), and rabbit anti-SOX9 antibody (Santa Cruz Biotechnology Inc., Dallas, Texas, United States). The quantification histological analysis was performed from six randomly selected areas per sample by three experienced investigators [22].

### 2.6. Statistical Analysis

Results were expressed as mean and standard deviation (*M* ± SD). To compare mean values within a group, between two groups, and across multiple groups, statistical analyses included paired and unpaired Student's *t*-tests and ANOVA. Kaplan–Meier survival analysis was performed to compare the time to onset of euglycemia between experimental groups. The proportion of mice remaining diabetic over time was plotted, and differences between groups were assessed using the log-rank (Mantel–Cox) test. A value of *p* < 0.05 was significant.

## 3. Results

### 3.1. Effects of Exendin-4 on the NPCC Differentiation Within the Graft

**Figure 1:**
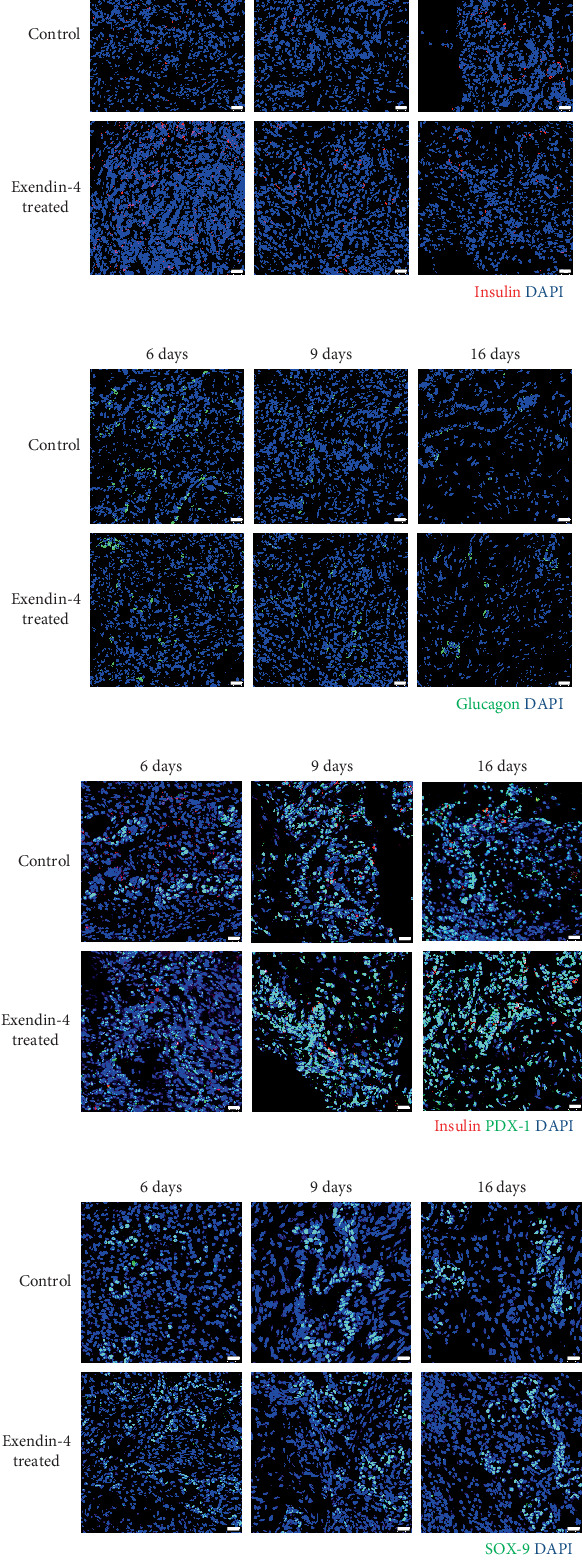
Immunofluorescence staining of graft sections for (a) insulin (red), (b) glucagon (green), (c) insulin (red) and PDX1 (green), and (d) SOX9 (green) in control (upper panels) and exendin-4-treated (lower panels) groups at 6, 9, and 16 days following transplantation of 2000 NPCCs. Cell nuclei are counterstained with DAPI (blue). Scale bar: 50 *μ*m.

**Figure 2:**
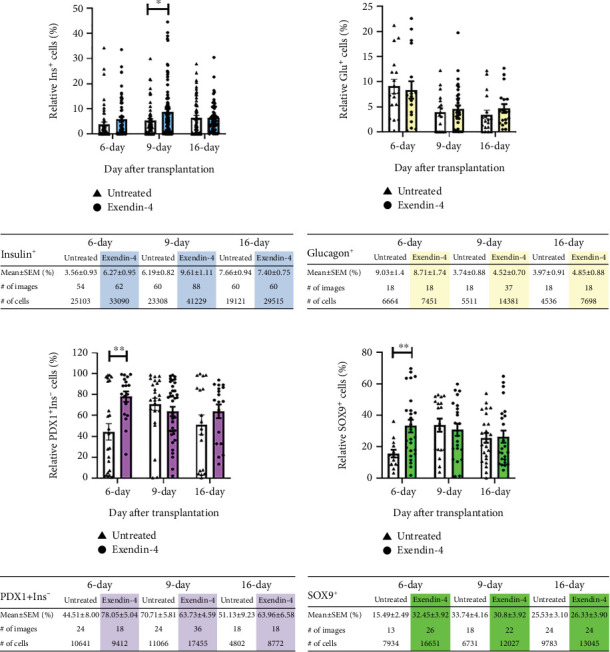
Quantitative analysis of immunofluorescence staining for (a) insulin^+^, (b) glucagon^+^, (c) PDX1^+^/insulin^−^, and (d) SOX9^+^ cells in grafts from control (open columns) and exendin-4-treated (colored columns) groups at 6, 9, and 16 days posttransplantation of 2000 NPCCs. Each data point represents a quantitative measurement obtained from a single randomly selected field of a tissue section at the specified time point. ⁣^∗^*p* < 0.05 and ⁣^∗∗^*p* < 0.01.

Our prior research demonstrated that, following NPCC transplantation, the proportion of mature insulin^+^ and glucagon^+^ cells increased at 9 and 16 days while PDX1^+^/insulin^−^ and SOX9^+^ progenitor cells declined at 6, 9, and 16 days in the grafts of diabetic mice [22]. Based on this, we subcutaneously injected exendin-4 (3 *μ*g/kg) or saline twice daily to diabetic nude mice transplanted with 2000 NPCCs. Grafts were harvested at Days 6, 9, and 16 (*n* = 9, 5, and 5 for the control group and *n* = 7, 9, and 8 in the exendin-4-treated group, respectively) to assess the NPCC differentiation via immunofluorescence staining for insulin, glucagon, PDX1, and SOX9. The results showed that exendin-4 significantly increased the proportion of insulin^+^ cells at Day 9 (*p* < 0.05), suggesting a transient promotion of NPCC differentiation. Additionally, at Day 6, the exendin-4 group showed a significant increase in both PDX1^+^/insulin^−^ and SOX9^+^ progenitor cells (*p* < 0.01), indicating an expansion of the progenitor pool (Figures 1 and 2). A similar trend was observed when comparing mean values per mouse at each time point; however, these differences did not reach statistical significance, likely due to the limited sample size (Figure S1).

### 3.2. Long-Term Effects of Exendin-4 on NPCC Recipients' Blood Glucose and Body Weight

**Figure 3:**
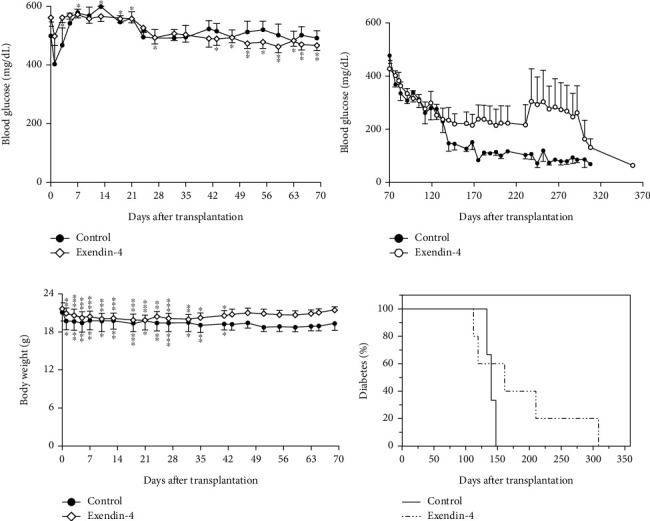
Time course of (a, b) nonfasting blood glucose levels and (c) body weight in diabetic nude mice after transplantation of 2000 NPCCs in each mouse with (open diamonds) and without (solid circles) exendin-4 treatment. Data are presented as mean ± SD. ⁣^∗^*p* < 0.05, ⁣^∗∗^*p* < 0.01, and ∗∗∗*p* < 0.001 versus Day 0. (d) The Kaplan–Meier survival curve depicting the proportion of diabetic mice over time in the exendin-4-treated group (dotted line, *n* = 5) and the control group (solid line, *n* = 3). *p* = 0.6547 between the two groups.

Following transplantation, NPCC recipients were treated with either exendin-4 or saline subcutaneously twice daily for up to 280 days. In the control group (*n* = 6), blood glucose levels increased over the first 3 weeks before returning to baseline. In contrast, the exendin-4-treated group (*n* = 13) maintained their baseline glucose levels initially, followed by a decline after 6 weeks. By 10 weeks, glucose levels remained elevated in controls (499 ± 81 to 491 ± 81 mg/dL, *p* = 0.7993), while a significant reduction was observed in the exendin-4 group (561 ± 51 to 466 ± 61 mg/dL, *p* = 0.0042). Notably, blood glucose levels in the exendin-4-treated mice were significantly lower than baseline starting from Week 6. However, the overall difference between the two groups was not statistically significant (Figure 3).

In both groups, body weight decreased during the first days, then stabilized and gradually returned to baseline after 6 weeks, with no significant differences observed between groups throughout the study (Figure 3c). In 10 recipients maintained over 10 weeks, two of exendin-4-treated mice remained to have hyperglycemia and died at 186 and 300 days, respectively; three control and five exendin-4-treated mice achieved euglycemia between 119 and 308 (mean ± SD: 166 ± 65) days (Figure 3b). The Kaplan–Meier analysis showed no significant difference in diabetes-free survival between groups (*p* = 0.6547) (Figure 3d).

### 3.3. Histological Analysis of Long-Term NPCC Grafts

**Figure 4:**
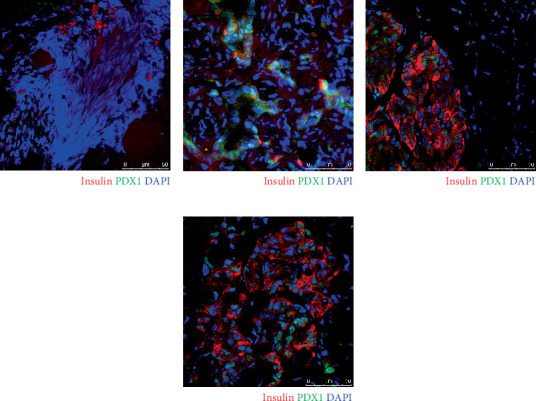
Immunofluorescence staining of insulin (red) and PDX1 (green) in grafts from control mice at (a) 70 days and (c) 302 days and from exendin-4-treated mice at (b) 70 days and (d) 358 days following transplantation of 2000 NPCCs. Cell nuclei are stained with DAPI (blue). Scale bar: 50 *μ*m.

At 10 weeks posttransplantation, despite persistent hyperglycemia in both groups, grafts contained substantial numbers of insulin^+^ cells (Figure 4a,b). Progenitor cells characterized by insulin^−^/PDX1^+^ expression were also observed (Figure 4b). In contrast, grafts from mice that achieved euglycemia displayed a prominent population of mature insulin^+^/PDX1^+^ cells, indicating maturation of *β*-cells (Figure 4c,d).

## 4. Discussion

A well-recognized challenge in NPCC transplantation is the extended delay before achieving glycemic normalization in diabetic nude mice [4–9]. Given the known effects of exendin-4 in stimulating *β*-cell replication and neogenesis [14, 15], as well as inhibiting *β*-cell apoptosis [15, 16], we hypothesized that exendin-4 might enhance NPCC differentiation and function in diabetic recipients. In this study, we first investigated whether posttransplant exendin-4 treatment could promote the differentiation of grafted NPCCs. Our earlier work demonstrated that the proportion of mature insulin^+^ and glucagon^+^ cells increased at Days 9 and 16, while SOX9^+^ progenitor cells declined at Days 6, 9, and 16 after NPCC transplantation in STZ-induced diabetic mice. Building on these findings, we assessed the impact of exendin-4 on NPCC graft differentiation by quantifying insulin^+^ and glucagon^+^ mature endocrine cells, as well as PDX1^+^/insulin^−^ and SOX9^+^ progenitor cells at the same time points. Our current data revealed that exendin-4 treatment led to a transient increase in insulin^+^ cells at Day 9, suggesting a potential role in promoting NPCC differentiation. This pattern differs from earlier studies that reported sustained enhancement of graft differentiation following exendin-4 treatment in diabetic recipients transplanted with immature mouse or human fetal islet-like cell clusters [20, 21]. Several factors may account for this discrepancy. First, NPCCs are inherently more heterogeneous and may undergo differentiation at a slower rate than mouse and human fetal islet-like clusters [22]. Second, the route of exendin-4 administration varied—those studies used intraperitoneal injection, whereas we employed subcutaneous delivery. Third, prior analyses focused solely on insulin^+^ cells at later stages (10 days and 3 months) posttransplantation, while our study evaluated a broader panel of markers—including insulin^+^, glucagon^+^, PDX1^+^/insulin^−^, and SOX9^+^ cells—at multiple early time points (Days 6, 9, and 16). Importantly, we also observed a significant increase in PDX1^+^/insulin^−^ and SOX9^+^ progenitor cells at Day 6 in exendin-4-treated grafts, implying that exendin-4 may expand the progenitor cell pool. Since progenitor-mediated neogenesis has been identified as a contributor to *β*-cell regeneration after NPCC transplantation [5, 22], this early enrichment of progenitor cells may underlie the transient increase in insulin^+^ cells at Day 9.

We also examined the long-term effects of exendin-4 on NPCC recipients. Although both control and exendin-4-treated groups remained hyperglycemic at 10 weeks, blood glucose levels in the exendin-4 group declined significantly from baseline beginning at Week 6. While exendin-4 induced only a transient increase in NPCC differentiation during the early posttransplant period, its glucose-lowering effect is likely mediated through its established physiological mechanisms, including stimulation of insulin secretion, suppression of glucagon release, delayed gastric emptying, appetite suppression, and enhanced peripheral glucose uptake [10]. The lack of significant difference in overall glycemic control between the two groups may be attributed to the higher baseline glucose levels in the exendin-4-treated group (561 ± 51 vs. 499 ± 81 mg/dL in controls). Euglycemia was achieved in three control and five exendin-4-treated mice between Days 119 and 308. These findings are consistent with earlier reports showing normoglycemia occurring between 9 and 16 weeks after NPCC transplantation [6]. At 10 weeks, grafts from hyperglycemic mice showed both insulin^−^/PDX1^+^ (progenitor cells) and insulin^+^/PDX1^+^ (mature *β*-cells), indicating ongoing differentiation. In contrast, normoglycemic recipients exhibited predominantly mature insulin^+^/PDX1^+^ cells, consistent with the morphological and functional maturation of their NPCC grafts.

We also evaluated the long-term impact of exendin-4 treatment on NPCC recipients. Although both the control and exendin-4-treated groups remained hyperglycemic at 10 weeks, a significant reduction in blood glucose levels was observed in the exendin-4 group starting from Week 6. This occurred despite exendin-4 only transiently enhancing NPCC differentiation during the early posttransplant period.

## 5. Conclusions

NPCCs offer a promising and accessible source for islet transplantation. This study demonstrates that exendin-4 transiently enhances NPCC differentiation and expands the progenitor cell population at early posttransplant stages. Additionally, exendin-4 treatment was associated with improved glycemic control beginning at Week 6. However, it did not shorten the time to normoglycemia nor improve diabetes-free survival, suggesting limited long-term benefit. These findings emphasize the need for strategies that more effectively drive NPCC differentiation and *β*-cell maturation to advance cell-based therapies for Type 1 diabetes.

## Figures and Tables

**Table 1:** Summary of the number of pigs and mice used in the study.

	**Groups**	**Short-term experiment (6, 9 and 16 days)**	**Long-term experiment (≥ 70 days)**
Pigs		12	4
Nude mice	Control group	Day 6 (*n* = 9) Day 9 (*n* = 5) Day 16 (*n* = 5)	*n* = 6
	Exendin-4-treated group	Day 6 (*n* = 7) Day 9 (*n* = 9) Day 16 (*n* = 8)	*n* = 13

## Data Availability

The data that support the findings of this study are available from the corresponding authors upon reasonable request.

## Supporting Information

Supporting Information Additional supporting information can be found online in the Supporting Information section. Figure S1: Quantitative analysis of immunofluorescence staining for (A) insulin^+^, (B) glucagon^+^, (C) PDX1^+^/insulin^−^, and (D) SOX9^+^ cells in grafts from control (open columns) and exendin-4-treated (colored columns) groups at 6, 9, and 16 days posttransplantation of 2000 NPCCs. Each data point represents a quantitative measurement obtained from a mouse at the specified time point.
