# Supplementary material for: Impact of Exendin-4 on the Differentiation and Function of Transplanted Porcine Neonatal Pancreatic Cell Clusters in Diabetic Nude Mice
Source: J Diabetes Res. 2025 Oct 17;2025:5847576. doi: 10.1155/jdr/5847576 (PMC12552079; doi:10.1155/jdr/5847576)
Supplement: Supporting Information — Additional supporting information can be found online in the Supporting Information section. Figure S1: Quantitative analysis of immunofluorescence staining for (A) insulin+, (B) glucagon+, (C) PDX1+/insulin−, and (D) SOX9+ cells in grafts from control (open columns) and exendin-4-treated (colored columns) groups at 6, 9, and 16 days posttransplantation of 2000 NPCCs. Each data point represents a quantitative measurement obtained from a mouse at the specified time point. [file 5847576.f1.docx]

# Supplementary

#
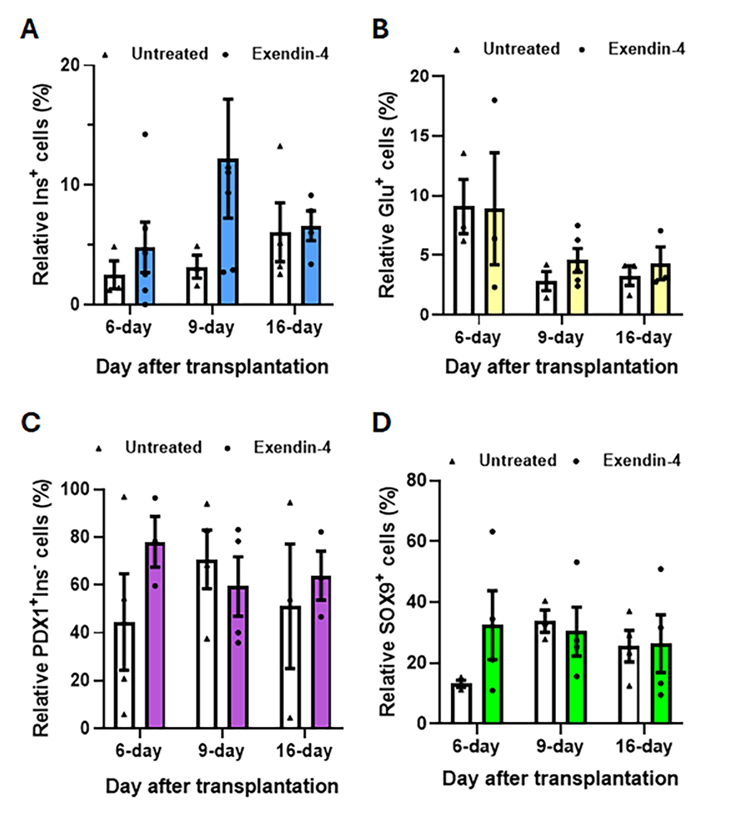


Figure S1: Quantitative analysis of immunofluorescence staining for (A) insulin^+^, (B) glucagon^+^, (C) PDX1^+^/insulin⁻, and (D) SOX9^+^ cells in grafts from control (open columns) and exendin-4 treated (colored columns) groups at 6, 9, and 16 days posttransplantation of 2000 NPCCs. Each data point represents a quantitative measurement obtained from a mouse at the specified time point.
